# Comparison of Use of Neoadjuvant Systemic Treatment for Breast Cancer and Short-term Outcomes Before vs During the COVID-19 Era in Ontario, Canada

**DOI:** 10.1001/jamanetworkopen.2022.25118

**Published:** 2022-08-02

**Authors:** Steven Habbous, Xiaochen Tai, Jaclyn M Beca, Jessica Arias, Michael J. Raphael, Ambica Parmar, Andrea Crespo, Matthew C Cheung, Andrea Eisen, Antoine Eskander, Simron Singh, Maureen Trudeau, Scott Gavura, Wei Fang Dai, Jonathan Irish, Monika Krzyzanowska, Lauren Lapointe-Shaw, Rohini Naipaul, Stuart Peacock, Lyndee Yeung, Leta Forbes, Kelvin K. W. Chan

**Affiliations:** 1 Ontario Health (Cancer Care Ontario), Toronto, Ontario, Canada; 2 Canadian Centre for Applied Research in Cancer Control, Toronto, Ontario, Canada; 3 Division of Medical Oncology, Sunnybrook Health Sciences Centre, Toronto, Ontario, Canada; 4 Hematology, Sunnybrook Health Sciences Centre, Toronto, Ontario, Canada; 5 Department of Otolaryngology–Head and Neck Surgery, University of Toronto, Toronto, Ontario, Canada; 6 Institute for Health Policy, Management and Evaluation, University of Toronto, Toronto, Canada; 7 Department of Medical Oncology & Hematology, Princess Margaret Cancer Centre, Toronto, Ontario, Canada; 8 Department of Medicine, University of Toronto, Toronto, Ontario, Canada; 9 Cancer Control Research, BC Cancer, Vancouver, British Columbia, Canada; 10 Division of Medical Oncology, RS McLaughlin Durham Regional Cancer Centre Lakeridge Health, Oshawa, Ontario, Canada

## Abstract

**Importance:**

In response to an increase in COVID-19 infection rates in Ontario, several systemic treatment (ST) regimens delivered in the adjuvant setting for breast cancer were temporarily permitted for neoadjuvant-intent to defer nonurgent breast cancer surgical procedures.

**Objective:**

To examine the use and compare short-term outcomes of neoadjuvant-intent vs adjuvant ST in the COVID-19 era compared with the pre–COVID-19 era.

**Design, Setting, and Participants:**

This was a retrospective population-based cohort study in Ontario, Canada. Patients with cancer starting selected ST regimens in the COVID-19 era (March 11, 2020, to September 30, 2020) were compared to those in the pre–COVID-19 era (March 11, 2019, to March 10, 2020). Patients were diagnosed with breast cancer within 6 months of starting systemic therapy.

**Main Outcomes and Measures:**

Estimates were calculated for the use of neoadjuvant vs adjuvant ST, the likelihood of receiving a surgical procedure, the rate of emergency department visits, hospital admissions, COVID-19 infections, and all-cause mortality between treatment groups over time.

**Results:**

Among a total of 10 920 patients included, 7990 (73.2%) started treatment in the pre–COVID-19 era and 7344 (67.3%) received adjuvant ST; the mean (SD) age was 61.6 (13.1) years. Neoadjuvant-intent ST was more common in the COVID-19 era (1404 of 2930 patients [47.9%]) than the pre–COVID–19 era (2172 of 7990 patients [27.2%]), with an odds ratio of 2.46 (95% CI, 2.26-2.69; *P* < .001). This trend was consistent across a range of ST regimens, but differed according to patient age and geography. The likelihood of receiving surgery following neoadjuvant-intent chemotherapy was similar in the COVID-19 era compared with the pre–COVID-19 era (log-rank *P* = .06). However, patients with breast cancer receiving neoadjuvant-intent hormonal therapy were significantly more likely to receive surgery in the COVID-19 era (log-rank *P* < .001). After adjustment, there were no significant changes in the rate of emergency department visits over time between patients receiving neoadjuvant ST, adjuvant ST, or ST only during the ST treatment period or postoperative period. Hospital admissions decreased in the COVID-19 era for patients who received neoadjuvant ST compared with adjuvant ST or ST alone (*P* for interaction = .01 for both) in either setting.

**Conclusions and Relevance:**

In this cohort study, patients were more likely to start neoadjuvant ST in the COVID-19 era, which varied across the province and by indication. There was limited evidence to suggest any substantial impact on short-term outcomes.

## Key Points

**Question:**

Did the use of neoadjuvant-intent systemic therapy for patients with breast cancer change after the emergence of COVID-19 in Ontario, Canada?

**Findings:**

In this cohort study including 10 920 patients, the use of neoadjuvant-intent chemotherapy and hormonal treatment increased during the COVID-19 era, but there was substantial regional variability. Bridging hormonal therapy was a more common adaptation to cancer treatment in the COVID-19 era than neoadjuvant chemotherapy; neoadjuvant-intent systemic treatment was not associated with short-term outcomes in the COVID-19 era.

**Meaning:**

These findings suggest that patients with breast cancer were more likely to receive neoadjuvant-intent systemic treatment in the COVID-19 era to offset delays in surgical capacity.

## Teaser

This cohort study investigates the use of and short-term outcomes associated with neoadjuvant-intent vs adjuvant systemic treatment for breast cancer in the COVID-19 era compared with the pre–COVID-19 era.

## Introduction

The COVID-19 pandemic changed the landscape of health care delivery. When infection rates began to rise in Ontario, concerns grew that patients with COVID-19 requiring respiratory support would reduce the capacity of intensive care units for non-COVID-19–related care, as had happened in other jurisdictions that experienced the first wave earlier than Canada (eg, Italy, New York).^1^ For patients with cancer, the effects that COVID-19 infection would have on their health status were largely unknown at the time. Given the immunocompromised state of many patients with cancer, the concern was that a COVID-19 infection might portend an even higher chance of morbidity and mortality in this patient population.^2,3^

These concerns led to a series of policy changes to alter the care of patients in Ontario and preempt the anticipated surge of patients requiring inpatient acute and critical care beds. To this effect, many nonurgent surgical procedures to treat cancer were postponed.^4,5,6^ Consequently, some patients with cancer who were eligible for primary surgical resection could be offered neoadjuvant systemic treatment (ST), with the intent that surgery would occur at a later date when hospital burden had subsided.

By the start of the first wave of the COVID-19 pandemic in Ontario, several ST regimens that previously were only publicly funded in the adjuvant setting were temporarily extended to the neoadjuvant setting.^7,8,9^ In the present study, we measured the use of publicly funded neoadjuvant-intent ST for patients with breast cancer, examined regional variability, and assessed whether any changes in treatment modality were associated with short-term patient outcomes.

## Methods

This was a retrospective, population-based cohort study conducted in Ontario, Canada. Analyses were conducted between June 2020 and November 2021, with all data extracted in November 2021. Research ethics approval was not required as per an Ontario Health (Cancer Care Ontario) privacy assessment. This study is reported as per the Strengthening the Reporting of Observational Studies in Epidemiology (STROBE) reporting guideline for cohort studies.

### Cohort Identification and Data Sources

Patients were adults (aged at least 18 years) who received ST between March 11, 2019, and September 30, 2020, for a new primary breast cancer. The ST regimens included were determined a priori based on existing or new ST protocols that may be provided in either the neoadjuvant or adjuvant settings and categorized as chemotherapy or hormonal therapy (eTable 1 in the Supplement).

In Ontario, most injectable cancer therapies (eg, intravenous chemotherapy) are funded by the Systemic Treatment-Quality Based Procedures (ST-QBP), identified from the Activity Level Reporting (ALR) database. ALR includes information on ST administered in ambulatory cancer clinics within hospitals across Ontario (99% of all ST administered in the province). Treatment regimen data reported via ALR are for purposes of hospital funding, regardless of funding source for individual drugs included in the regimen. Many oral or take-home cancer drugs (eg, hormones), although sometimes funded by the Ontario Drug Benefit program, are captured by the ALR system when prescriptions are entered using the in-hospital Computerized Provider Order Entry system.^10^ Treatment intent is indicated by the most responsible physician (typically a medical oncologist or hematologist) using the Computerized Provider Order Entry system.

### Cohort Exclusions

Patients were excluded if any ST visit was coded with palliative intent. To ensure patients were receiving treatment for a primary malignant neoplasm, the date of the most recent invasive cancer diagnosis preceding the start of ST was obtained from the Ontario Cancer Registry (OCR). To ensure only ST for the primary disease was captured, patients diagnosed more than 6 months before starting ST were omitted (eFigure 1 in the Supplement).

### Data Sources for Surgery and Cohort Subclassification

To classify patients as having received neoadjuvant ST, adjuvant ST, or ST-only, we used the surgery date obtained from the Wait Time Information System (WTIS). The first resection corresponding to the patients’ ST regimen on or after the OCR diagnosis date was used. If the surgery date occurred between the diagnosis date and the start of ST (inclusive), the patient was categorized as adjuvant ST. If the resection occurred within 12 months after the start of ST, then the patient was classified as neoadjuvant ST. Twelve months was chosen because this was a reasonable upper limit to the time required to complete a standard ST regimen and proceed to surgery, even with delays due to COVID-19. Furthermore, most surgical procedures were captured during this window (eFigure 2 in the Supplement). If no surgery date was identified, then the patient was classified as having received ST-only.

Patients were assigned to the pre–COVID-19 era if they started treatment (surgery or ST) between March 11, 2019, and March 10, 2020 (the day before Ontario’s Ministry of Health announced funding policy changes due to COVID-19). If the patient received surgery or ST before March 11, 2019, then the patient was excluded. Patients were assigned to the COVID-19 era if their first treatment occurred between March 11, 2020, and September 30, 2020 (the putative end of the first wave of COVID-19 in Ontario).

### Outcomes

**Figure 1.**
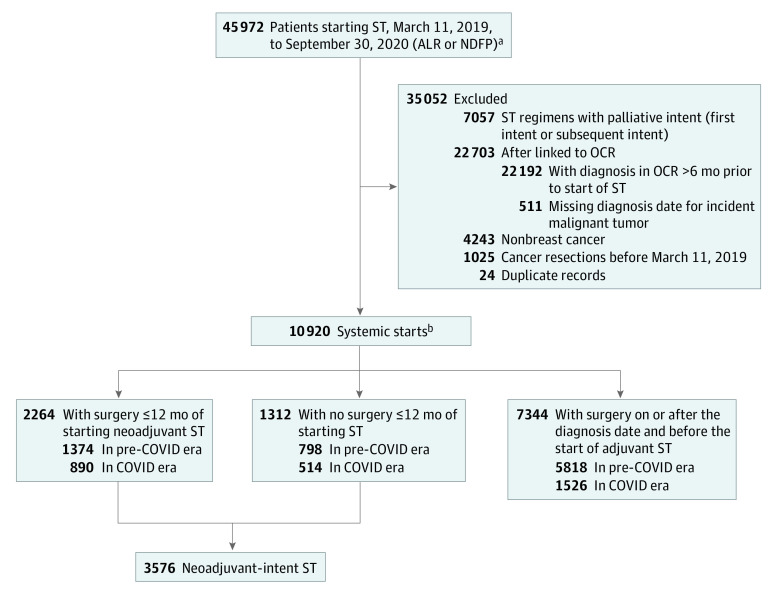
Patient Inclusion and Exclusion ALR indicates Activity Level Reporting database; NDFP, New Drug Funding Program database; OCR, Ontario Cancer Registry; and ST, systemic therapy. ^a^ST start was identified as the date of ST following a gap of no ST until January 1st. ^b^Eight patients started treatment in both eras for 2 distinct primary cancer diagnoses.

The primary outcome was receipt of neoadjuvant-intent vs adjuvant ST in the COVID-19 era vs the pre–COVID-19 era. Patients receiving neoadjuvant-intent ST received either ST-only or ST followed by surgery (Figure 1). Secondary outcomes included transition to surgery within 12 months after starting neoadjuvant-intent ST and the rate of unscheduled emergency department (ED) visits and hospital admissions during the ST period and postoperative period. The ST period was defined as the time from the first ST visit until the earliest of surgery, death, or the last ST visit plus 30 days, whichever came first. The postoperative period was defined as the time from surgery until the first ST visit, death, 30 days after surgery, whichever came first. Visit rates were reported as the number of visits per patient-month and identified from the National Ambulatory Care Reporting System (ED visits) and the Discharge Abstract Database (admissions). By linking to the Ontario Laboratory Information System, we also estimated the proportion of patients during the COVID-19 era having at least 1 positive COVID-19 test, up until September 30, 2021. Lastly, we estimated the number of patients who died using the OCR and the Registered Persons Database. Mortality data were available until December 31, 2021. The follow-up period for mortality started from the date of surgery for patients receiving neoadjuvant ST or the start of ST for patients receiving ST-only or adjuvant ST (to remove immortal time bias).

### Regional Variability

We explored whether there was regional variability in the primary outcome. The geographical unit of analysis was the Local Health Integration Network (LHIN). For correlation of the primary outcome with local COVID-19 infection rates per capita, the number of COVID-19 infections per Public Health Unit (PHU) between March 2020 and September 2020 was obtained from publicly available data (accessed from the Ontario Data Catalogue on November 3, 2021) and corresponding population counts.^11,12^

### Statistical Analysis

To evaluate whether patients in the COVID-19 era were more likely than patients in the pre–COVID-19 era to receive neoadjuvant-intent ST vs adjuvant ST, we used logistic regression, presenting odds ratios (ORs) with 95% CIs. We also explored whether sociodemographic characteristics were associated with neoadjuvant-intent ST, and using interaction terms, whether these associations differed in the pre–COVID-19 and COVID-19 eras. Sociodemographic characteristics included age when treatment started, sex, rurality, and neighborhood-level median after-tax household income quintile using the Postal Code Conversion File (PCCF+) version 7C (2016 Census).

To evaluate whether patients receiving neoadjuvant-intent ST were more or less likely to proceed to surgery in the COVID-19 era vs the pre–COVID-19 era, we used a time-to-event analysis with surgery as the event. Patients were censored after 12 months, death, or the end of the study follow-up (December 31, 2021), whichever came first. Results were presented descriptively using cumulative incidence plots. Hazard ratios (HRs) with 95% CIs were only reported when violations of the proportionality assumption were believed to be small by visual inspection of Kaplan-Meier plots and ln(−ln[survival]) plots. These analyses were unadjusted because the choice of treatment as neoadjuvant-intent or adjuvant ST is likely confounded by multiple unmeasured and unavailable confounders, such as stage. Differences in ED visit rates or admission rates were evaluated using Poisson regression with the natural logarithm of the follow-up time as the offset to account for different follow-up times between groups. Exponentiated coefficients are interpreted as rate ratios (RR). These analyses were adjusted for sociodemographic characteristics, geography, and disease site. All-cause mortality and COVID-19 positivity were descriptive analyses and were unadjusted.

All analyses were conducted at Ontario Health using SAS version 9.4 (SAS Institute) and cumulative incidence plots using R version 1.2.5033 (R Project for Statistical Computing). Results were considered statistically significant at a 2-tailed *P* < .05.

## Results

After exclusions, 10 920 patients received ST for a new breast cancer diagnosis between March 11, 2019, and September 30, 2020 (Figure 1). A total 5493 patients (50.3%) received chemotherapy first, and 5427 patients (49.7%) received hormonal ST first. Patients had a mean (SD) age of 61.6 (13.1) years, 9644 (88.3%) lived in an urban neighborhood, and 7344 (67.3%) received adjuvant ST, 2264 (20.7%) received neoadjuvant ST, and 1312 (12.0%) received ST alone. Patients in the pre–COVID-19 era and COVID-19 era were similar with respect to mean (SD) age (61.9 [12.8] vs 60.6 [13.7] years), sex (99.2% [7925 of 7990] vs 99.0% [2902 of 2930]), neighborhood income quintile (eg, lowest-income quartile: 17.1% [1356 of 7990] vs 17.0% [495 of 2930]) were in the lowest-income quintile in both eras), and rurality (eg, urban neighborhood: 88.8% [7037 of 7990] vs 89.5% [2607 of 2930]) (Table 1). Patients were more likely to receive neoadjuvant-intent ST in the COVID-19 era (1404 patients 47.9%) than the pre–COVID era (2172 patients [27.2%]) (OR, 2.46; 95% CI, 2.26-2.69; *P* < .001).

**Table 1.** Patient Demographics

Characteristic	Patients, No. (%)^a^	
	Pre–COVID-19 era (n = 7990)	COVID-19 era (n = 2930)
Age at start of systemic therapy, mean (SD), y	61.9 (12.8)	60.6 (13.7)
Sex		
Female	7925 (99.2)	2902 (99.0)
Male	65 (0.8)	28 (1.0)
After-tax median household income quintile^b^		
Highest	1753 (22.1)	702 (24.1)
Mid-high	1682 (21.3)	615 (21.1)
Middle	1562 (19.7)	554 (19.0)
Mid-low	1565 (19.8)	546 (18.8)
Lowest	1356 (17.1)	495 (17.0)
Rurality^b^		
Urban	7037 (88.8)	2607 (89.5)
Rural	886 (11.2)	305 (10.5)
Systemic treatment start		
Adjuvant setting	5818 (72.8)	1526 (52.1)
Neoadjuvant setting	1374 (17.2)	890 (30.4)
Systemic alone	798 (10.0)	514 (17.5)
Systemic treatment indication		
Breast (chemotherapy)	3894 (48.7)	1599 (54.6)
Breast (hormonal therapy)	4096 (51.3)	1331 (45.4)

^a^ Patients started treatment with surgery or systemic therapy between March 11, 2019 and March 10, 2020 (pre–COVID era) or March 11, 2020 and September 30, 2020.

^b^ Source (or adapted from): Statistics Canada Postal Code Conversion File and Postal Code Conversion File Plus (received August 2020) which is based on data licensed from Canada Post Corporation (version 7C). The patients’ postal code at diagnosis was used.

Patients receiving neoadjuvant-intent ST were younger the pre–COVID era (OR, 0.78 ; 95% CI, 0.75-0.81) and the COVID era (OR, 0.86; 95% CI, 0.82-0.91), but this association was attenuated in the COVID-19 era (*P* for interaction = .003). Conversely, there was no association between receiving neoadjuvant-intent ST and the patients’ neighborhood income quintile or rurality).

### Indication

Patients were more likely to receive neoadjuvant-intent ST in the COVID-19 era than the pre–COVID era for both hormonal therapy (OR, 3.17; 95% CI, 2.75-3.65; *P* < .001) and chemotherapy (OR, 2.07; 95% CI, 1.84-2.33; *P* < .001) (eTable 2 and eFigure 3 in the Supplement).

### Regional Variability

**Figure 2.**
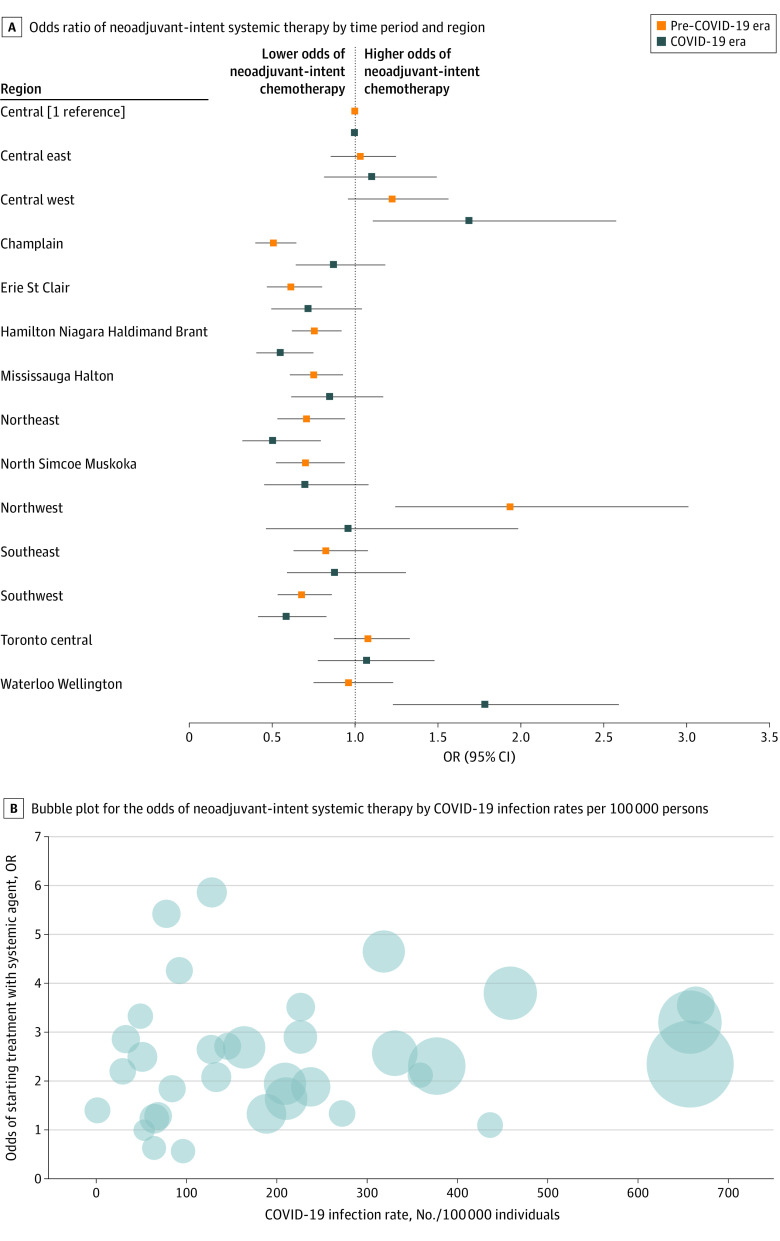
Regional Variation Likelihood of Providing Neoadjuvant-Intent Systemic Therapy

Receipt of neoadjuvant-intent ST differed across regions between eras (*P* for interaction < 0.001) (Figure 2A). Stratified by LHIN (eTable 3 in the Supplement), receipt of neoadjuvant-intent chemotherapy was either as likely (OR, 1.04; 95% CI, 0.44-2.43) or more likely (OR, 3.73; 95% CI, 2.25-6.18) in the COVID-19 era vs the pre–COVID-19 era across regions. In contrast, neoadjuvant-intent hormonal therapy was significantly more likely in the COVID-19 era across all LHINs, with ORs ranging from 1.84 (95% CI, 1.03-3.28) to 6.86 (95% CI, 4.00-11.77). Using the public health unit as the geographical unit (eTable 4 in the Supplement), we found a correlation between the odds of neoadjuvant-intent ST in the COVID era with local COVID-19 infection rates per capita (Pearson *r* = 0.150; Figure 2B).

### Patient Outcomes: Receipt of Surgery

**Figure 3.**
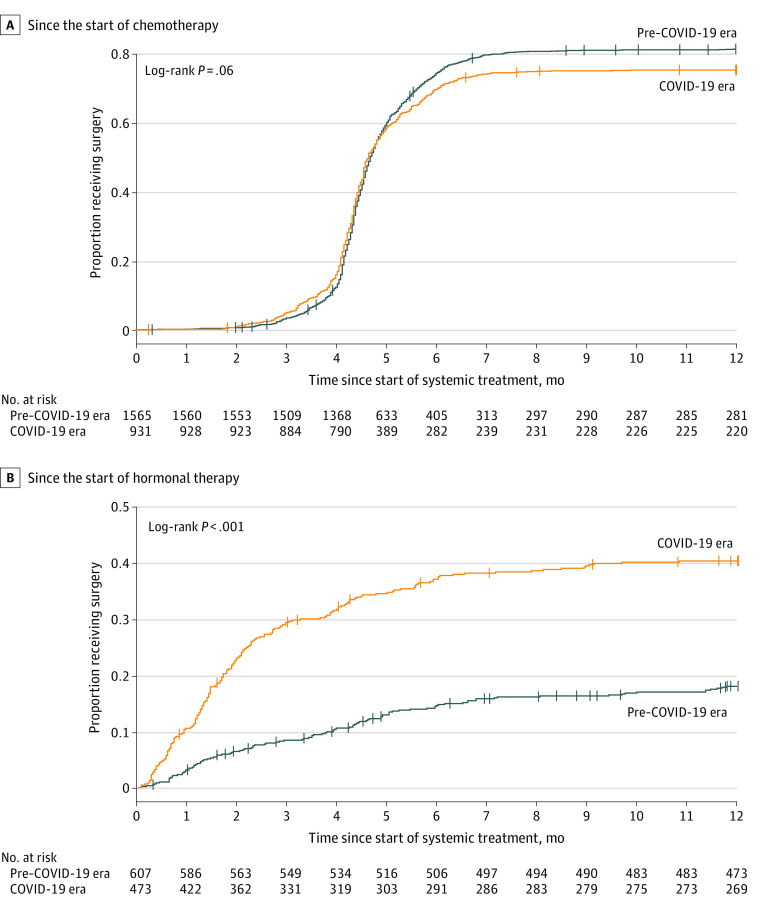
Cumulative Incidence Plots of the Likelihood of Receiving Surgery Over Time Since the Start of Chemotherapy or Hormonal Therapy for Patients With Breast Cancer

Among patients receiving neoadjuvant-intent breast chemotherapy, receipt of surgery followed a similar pattern between the pre–COVID-19 and COVID-19 eras, increasing sharply at the time chemotherapy is expected to have been completed (14-18 weeks after starting chemotherapy) and leveling off after 6 months (Figure 3A). By the end of follow-up, 81% and 75% of patients subsequently received surgery in the pre–COVID-19 and COVID-19 eras, respectively (log-rank *P* = .06). In contrast, patients who received neoadjuvant-intent hormonal treatment in the COVID-19 era were significantly more likely to receive subsequent surgery than in the pre–COVID-19 era (HR, 3.17; 95% CI, 2.75-3.65; log-rank *P* < .001) (Figure 3B).

### Emergency Department Visits and Hospital Admissions

The rate of emergency department (ED) visits decreased from 14.8 (95% CI, 13.6-16.0) to 12.5 (95% CI, 11.2-13.8) visits per 100 person-months for patients receiving neoadjuvant ST in the pre–COVID-19 and COVID-19 eras, respectively (Table 2). Conversely, the ED visit rate increased for patients receiving adjuvant ST (6.7 95% CI, 6.4-7.0] to 8.8 [95% CI, 8.0-9.7]) visits per 100 patient-months) or ST-only (8.0 [95% CI, 7.1-8.9] to 8.9 [95% CI, 7.6-10.5] visits per 100 patient-months). This difference was statistically significant (*P* < .001 for interaction between treatment type and era). After adjustment for age, disease site, income quintile, rurality, treatment type, and LHIN, there was no difference in eras for ED visit rate between patients receiving neoadjuvant ST, adjuvant ST, and ST-only (*P* for interaction = .15). Similarly, ED visits during the postoperative phase did not differ by adjuvant or neoadjuvant ST over time (*P* for interaction = .17). In contrast, the reduction in hospital admissions during the ST period for patients receiving neoadjuvant ST (7.65 visits per person-month in the pre–COVID-19 era vs 6.31 visits per person-month in the COVID era) was significantly different from the increases over time observed for patients receiving adjuvant ST or ST-only (*P* for interaction = .01). Postoperative hospital admissions for patients receiving neoadjuvant ST were also lower in the COVID-19 era than the pre–COVID-19 era (2.66 vs 1.96 visits per patient-month) than that for patients receiving adjuvant ST (1.15 vs 1.22 visits per patient-month) (*P* for interaction = .01).

**Table 2.** Secondary Outcomes

Outcome^a^	Treatment group	No. patients at risk	No. visits, mean (SD)	Time at risk, mean (SD), mo	Rate per patient per month, mean (95% CI) × 100^b^	*P* value (Poisson regression)	Rate (adjusted) per patient per month, mean (95% CI) × 100^b^^,^^c^	*P* value (Poisson regression)
Unplanned ED visits during ST period^d^								
Pre–COVID-19	Neoadjuvant ST	1374	0.69 (1.08)	4.69 (3.25)	14.79 (13.71-15.97)	Era: <.001	22.3 (19.12-26.01)	Era: .44
	ST only	798	0.53 (1.07)	6.64 (6.93)	7.97 (7.10-8.94)	Treatment: <.001	23.34 (19.6-27.8)	Treatment: <.001
	Adjuvant ST	5818	0.44 (1.00)	6.65 (6.32)	6.67 (6.37-6.98)	Interaction: <.001	19.49 (16.85-22.55)	Interaction: .15
COVID-19	Neoadjuvant ST	890	0.92 (1.13)	4.47 (2.93)	12.45 (11.20-13.84)	NA	20.15 (17.03-23.84)	NA
	ST only	514	0.41 (0.83)	4.62 (3.77)	8.93 (7.59-10.5)	NA	21.13 (17.2-25.96)	NA
	Adjuvant ST	1526	0.40 (0.96)	4.58 (3.66)	8.78 (7.98-9.66)	NA	20.27 (17.2-23.89)	NA
Hospital admissions during ST period^d^								
Pre–COVID-19	Neoadjuvant ST	1374	0.25 (0.56)	4.69 (3.25)	5.24 (4.82-5.7)	Era: <.001	7.65 (6.27-9.33)	Era: .35
	ST only	798	0.17 (0.46)	6.64 (6.93)	2.55 (2.23-2.91)	Treatment: <.001	6.52 (5.2-8.17)	Treatment: <.001
	Adjuvant ST	5818	0.10 (0.38)	6.65 (6.32)	1.57 (1.47-1.67)	Interaction: <.001	4.09 (3.37-4.98)	Interaction: .01
COVID-19	Neoadjuvant ST	890	0.18 (0.49)	4.47 (2.93)	4.12 (3.65-4.65)	NA	6.31 (5.08-7.83)	NA
	ST only	514	0.15 (0.45)	4.62 (3.77)	3.33 (2.80-3.96)	NA	7.47 (5.81-9.61)	NA
	Adjuvant ST	1526	0.09 (0.35)	4.58 (3.66)	2.06 (1.81-2.35)	NA	4.38 (3.51-5.47)	NA
Unplanned ED visits during postoperative period^e^								
Pre–COVID-19	Neoadjuvant ST	1374	0.18 (0.54)	0.99 (0.00)	18.17 (16.40-20.12)	Era: <.001	24.51 (19.68-30.53)	Era: <.001
	Adjuvant ST	5818	0.16 (0.49)	0.84 (0.11)	16.56 (15.70-17.46)	Treatment: .12	23.44 (19.06-28.84)	Treatment: .48
COVID-19	Neoadjuvant ST	890	0.12 (0.42)	0.99 (0.00)	12.20 (10.44-14.25)	Interaction: .12	16.7 (13.03-21.41)	Interaction: .17
	Adjuvant ST	1526	0.12 (0.41)	0.91 (0.16)	13.30 (11.81-14.97)	NA	18.72 (14.83-23.63)	NA
Hospital admissions during postoperative period^e^								
Pre–COVID-19	Neoadjuvant ST	1374	0.03 (0.21)	0.99 (0.00)	3.47 (3.10-3.88)	Era: .62	2.66 (1.96-3.6)	Era: .50
	Adjuvant ST	5818	0.02 (0.13)	0.94 (0.11)	1.73 (1.60-1.87)	Treatment: <.001	1.15 (0.85-1.55)	Treatment: <.001
COVID-19	Neoadjuvant ST	890	0.02 (0.16)	0.99 (0.00)	2.51 (2.13-2.96)	Interaction: .01	1.96 (1.41-2.71)	Interaction: .01
	Adjuvant ST	1526	0.02 (0.13)	0.91 (0.16)	1.81 (1.55-2.11)	NA	1.22 (0.87-1.69)	NA

Abbreviations: NA, not applicable; ST, systemic treatment.

^a^ Patients started treatment in the pre–COVID-19 era (March 11, 2019, to March 10, 2020) or the COVID-19 era (March 11, 2020, to September 30, 2020).

^b^ *P* value from Poisson regression adjusted for the natural logarithm of the length of the ST period or postoperative period. The *P* value for era is the effect of the time period (COVID-19 era vs pre–COVID-19 era) on the outcome. The *P* value for treatment is the effect of the treatment group on the outcome. Lastly, the *P* value for the interaction corresponds to the effect of the era on the outcome based on the presence of treatment group (or vice versa).

^c^ Adjusted for age when treatment started (centered at age 62 years), income quintile (lowest quintile referent), rurality (rural residence referent), disease site group (breast chemotherapy referent), and Local Health Integration Network (Central LHIN referent). Rates are interpreted as the value for a patient age 62 with the referent value for all covariates.

^d^ The ST period was defined as the time from the first ST visit until the earliest of surgery, date of death, or the last ST visit plus 30 days, whichever came first.

^e^ The postoperative period was defined as the time from surgery until the first ST visit, date of death, or surgery plus 30 days, whichever came first.

Patients receiving neoadjuvant hormones were 77% less likely to visit the ED (risk ratio [RR], 0.23; 95% CI, 0.21-0.25; *P* < .001) or be admitted during the ST period (RR, 0.23; 95% CI, 0.21-0.26; *P* < .001). Patients receiving neoadjuvant hormones were 20% less likely to visit the ED during the postoperative period (RR, 0.80; 95% CI, 0.73-0.88 *P* < .001), but more likely to be admitted (RR, 1.16; 95% CI, 1.02-1.33), *P* = .03].

### COVID-19 Positivity and All-Cause Mortality

For patients starting treatment (surgery or ST) in the COVID-19 era, 41 patients (4.6%) receiving neoadjuvant ST had at least 1 COVID-19-positive test, which was higher than patients receiving adjuvant ST (32 patients; [2.1%]) or ST-only (12 patients [2.3%]) (χ^2^_2_ = 13.28; *P* = .001). Patients were more likely to contract COVID-19 if they started their cancer treatment after June 30, 2020 (eFigure 4 in the Supplement). Among patients who received neoadjuvant ST, 7% had a hospital admission within 14 days of the COVID-19-positive test, compared with 19% who received adjuvant ST and 25% who received ST-only, a total 7%, 19%, and 25% (Fisher *P* = .16).

All-cause mortality within 15 months was similar in the pre–COVID-19 era as the COVID-19 era for patients receiving adjuvant ST (1.1% vs 1.3%), ST-only (6.8% vs 4.9%), and neoadjuvant ST (3.5% vs 2.6%) (eFigure 5 in the Supplement).

## Discussion

In this study, we observed greater use of neoadjuvant-intent ST in the COVID-19 era compared with the pre–COVID-19 era for breast cancer patients receiving ST within 6 months of diagnosis. There was significant regional variation in the use of neoadjuvant-intent ST that was not associated with regional COVID-19 infection rates.

In response to COVID-19, the use of neoadjuvant ST increased in the United States,^13^ Brazil,^14^ Turkey,^15^ and elsewhere.^16^ In Australia, where there was a low burden of COVID-19 at the time, any changes in the dispensation of chemotherapy was small and transitory, driven by immunotherapies and targeted therapies.^17^ In contrast, the number of registrations for neoadjuvant-intent ST decreased in England and Italy due to COVID-19.^18,19^ In the United Kingdom, following multidisciplinary consultation many patients with breast cancer had incomplete neoadjuvant chemotherapy or instead received adjuvant chemotherapy.^20^ More frequent use of neoadjuvant endocrine therapy for breast cancer patients was reported from the United Kingdom and the United States with substantial regional variability (similar to our findings).^20,21^ Although not all cancers are suitable for neoadjuvant ST, substantial attention has been placed on breast cancer because of the array of biomolecular subtypes that can be used to identify patients who may have at least a neutral response to delaying surgery.^22,23^ For example, patients with hormone receptor-positive/ERBB2-negative stage I-III breast cancer can be treated with neoadjuvant endocrine therapy for 6 to 12 months before receiving surgery.^13,24^ Although we do not have timely data on stage and molecular subtype, our results suggest that the population of patients with breast cancer receiving neoadjuvant-intent hormonal agents in the pre–COVID-19 era differed from that of the COVID-19 era. These hormone-first patients were more likely to receive surgery in the COVID-19 era than the pre–COVID-19 era, suggesting that this group contains a subpopulation of patients whose treatment was truly being temporized due to COVID-19. In the pre–COVID-19 era, neoadjuvant hormones were not typically used unless the tumor was deemed unresectable.^25^ In the COVID-19 era, however, operating room availability was limited, so patients who otherwise would have received adjuvant hormones instead received bridging hormonal therapy for a few months before surgery. ED visits and hospitalization rates were 77% higher during the ST period for patients receiving chemotherapy compared with hormonal therapy. This may be due to greater toxicity profiles associated with chemotherapy. Future work should examine whether the immune suppression associated with cytotoxic chemotherapy increased the risk of developing symptomatic COVID-19 infection.

Understanding the medical and nonmedical factors that contribute to treatment decisions include patient choice and anxiety around COVID-19 rather than optimal medical management.^26,27^ Patients may be selected for neoadjuvant-intent ST in the COVID-19 era because the tumors were considered low-risk or potentially responsive to neoadjuvant ST. Conversely, this group may also include the most vulnerable patients, having some preexisting comorbidity that may increase the potential harms of a health care–acquired COVID-19 infection. Varied use of neoadjuvant-intent ST by geography may be driven by physician preferences, local (rather than regional) COVID-19 positivity rates, and perceived burden on hospital volumes. Efforts should be undertaken to establish whether harm was done (if neoadjuvant-intent treatment was suboptimal) in order to avoid this in the future. Alternatively, if neoadjuvant therapy abrogated the need for surgery without harm, further study is indicated to possibly expand treatment options to include this strategy. Our observation that COVID-19 infection rates were higher for patients receiving neoadjuvant ST was unexpected and contradicts the primary intent for introducing neoadjuvant ST as a treatment option. This may be due to a variety of medical and patient factors that are unavailable for study, but the lower rate of hospital admissions in this group after contracting COVID-19 is reassuring.

### Limitations

One limitation is misclassifying patients as receiving curative-intent treatment, particularly among the ST-only subgroup. However, we expect such misclassification to be non-differential between the periods. Moreover, we assume that all surgical procedures were performed with curative intent, but it is also possible that the type of surgery performed in the COVID-19 era differed from the pre–COVID-19 era. Another source of misclassification is the time point chosen to separate the pre–COVID-19 era from the COVID-19 era. Treatments starting shortly after this date likely follow treatment plans that originated in the pre–COVID-19 era. We expect this to shift the association of the COVID-19 era on the odds of providing neoadjuvant-intent ST toward unity. Another limitation is the potential for missing data on oral ST, particularly hormonal therapy. Although ALR captures many of these treatments, some may be missed. Another limitation is unavailability of timely staging and biomarker data. These data would be important to explore the potential misclassification of palliative-intent patients, and also to contextualize the findings in the face of possible stage migration.^28^ Lastly, patients who did not start therapy (surgery or ST) within 6 months of diagnosis due to avoidance of health care were not included. This represents a distinct population of patients whose outcomes and treatment may differ from those included in this study.

## Conclusions

We observed a higher proportion of patients receiving neoadjuvant-intent ST within 6 months of cancer diagnosis in Ontario during the COVID-19 era. Although there were no substantial detrimental short-term outcomes, whether these deviations in standard-of-care treatment influence long-term patient outcomes remains to be seen.

## Notes

Supplement. **eTable 1.** Systemic Therapy Regimens Included in the Study by Funding Source **eTable 2.** Number of Patients Receiving Each Systemic Therapy Regimen by Time Period and Type of Treatment **eTable 3.** Likelihood of Starting Treatment With A Systemic Agent by Region **eTable 4.** Likelihood of Starting Treatment With A Systemic Agent by Public Health Unit **eFigure 1.** The Distribution of Times (Months) From the Ontario Cancer Registry Diagnosis Date Until the First Systemic Treatment Date **eFigure 2.** The Distribution of Times (Days) From the Start of Systemic Treatment Until Receipt of Surgery for Patients Receiving Systemic Treatment First **eFigure 3.** Relative Odds of Starting Treatment With Systemic Treatment in the COVID Era Versus the Pre–COVID Era With Non-Palliative Intent **eFigure 4.** Risk of COVID Infection by Patient Group **eFigure 5.** All-Cause Mortality by Patient Group
