# Supplementary material for: Comparison of Use of Neoadjuvant Systemic Treatment for Breast Cancer and Short-term Outcomes Before vs During the COVID-19 Era in Ontario, Canada
Source: JAMA Netw Open. 2022 Aug 2;5(8):e2225118. doi: 10.1001/jamanetworkopen.2022.25118 (PMC9346546; doi:10.1001/jamanetworkopen.2022.25118)
Supplement: Supplement. — eTable 1. Systemic Therapy Regimens Included in the Study by Funding Source eTable 2. Number of Patients Receiving Each Systemic Therapy Regimen by Time Period and Type of Treatment eTable 3. Likelihood of Starting Treatment With A Systemic Agent by Region eTable 4. Likelihood of Starting Treatment With A Systemic Agent by Public Health Unit eFigure 1. The Distribution of Times (Months) From the Ontario Cancer Registry Diagnosis Date Until the First Systemic Treatment Date eFigure 2. The Distribution of Times (Days) From the Start of Systemic Treatment Until Receipt of Surgery for Patients Receiving Systemic Treatment First eFigure 3. Relative Odds of Starting Treatment With Systemic Treatment in the COVID Era Versus the Pre–COVID Era With Non-Palliative Intent eFigure 4. Risk of COVID Infection by Patient Group eFigure 5. All-Cause Mortality by Patient Group [file jamanetwopen-e2225118-s001.pdf]

Supplemental Online Content

Habbous S, Tai X, Beca JM, et al. Comparison of use of neoadjuvant systemic treatment for breast cancer and short-term outcomes before vs during the COVID-19 era in Ontario, Canada. *JAMA Netw Open.* 2022;5(8):e2225118. doi:10.1001/jamanetworkopen.2022.25118

eTable 1. Systemic Therapy Regimens Included in the Study by Funding Source

eTable 2. Number of Patients Receiving Each Systemic Therapy Regimen by Time Period and Type of Treatment

eTable 3. Likelihood of Starting Treatment With A Systemic Agent by Region

eTable 4. Likelihood of Starting Treatment With A Systemic Agent by Public Health Unit

eFigure 1. The Distribution of Times (Months) From the Ontario Cancer Registry Diagnosis Date Until the First Systemic Treatment Date

eFigure 2. The Distribution of Times (Days) From the Start of Systemic Treatment Until Receipt of Surgery for Patients Receiving Systemic Treatment First

eFigure 3. Relative Odds of Starting Treatment With Systemic Treatment in the COVID Era Versus the Pre-COVID Era With Non-Palliative Intent

eFigure 4. Risk of COVID Infection by Patient Group

eFigure 5. All-Cause Mortality by Patient Group

This supplemental material has been provided by the authors to give readers additional information about their work.

© 2022 Habbous S et al. *JAMA Network Open.*

eTable 1: Systemic therapy regimens included in the study by funding source

Regimen Code	Ontario Funder			Setting[a]	
	New Drug Funding Program	Systemic Treatment Quality-Based Procedures	Other Funder, if any	Pre-COVID era	COVID era
**Breast**					
AC-PACL(DD or W) +/-PERT + TRAS	Paclitaxel[c], trastuzumab	Doxorubicin and cyclophosphamide (AC)	Pertuzumab (none)	(neo)adjuvant	(neo)adjuvant
AC-PACL(DD or W) +/-TRAS	Paclitaxel[c], trastuzumab	Doxorubicin and cyclophosphamide (AC)		(neo)adjuvant	(neo)adjuvant
ANAS	None	None	Anastrozole (ODB-GB)	Adjuvant	(neo)adjuvant
CAPE	None	None	Capecitabine (ODB-GB)	Adjuvant	(neo)adjuvant
CMF(PO)	None	Fluorouracil, methotrexate	Cyclophosphamide (ODB-GB)	Adjuvant	(neo)adjuvant
CRBPDOCE+PERT+TRAS	Trastuzumab	Carboplatin, docetaxel	Pertuzumab (none)	Adjuvant	(neo)adjuvant
CRBPDOCETRAS	Trastuzumab	Carboplatin, docetaxel		(neo)adjuvant	(neo)adjuvant
CYCLDOCE; CYCLDOCE+TRAS[b]	Docetaxel, trastuzumab	Cyclophosphamide	None	Adjuvant	(neo)adjuvant
EXEM	None	None	Exemestane (ODB)	Adjuvant	(neo)adjuvant
FEC-D+PERT+TRAS	Docetaxel, epirubicin, trastuzumab	Fluorouracil, cyclophosphamide	Pertuzumab (none)	Adjuvant	(neo)adjuvant
FEC-D+/-TRAS	Docetaxel, epirubicin, trastuzumab	Fluorouracil, cyclophosphamide		(neo)adjuvant	(neo)adjuvant
GOSE	None	None	Goserelin (ODB-GB)	Adjuvant	(neo)adjuvant
KADC	None	None	Trastuzumab emtansine (CA)	Adjuvant	(neo)adjuvant
LETR	None	None	Letrozole (ODB-GB)	Adjuvant	(neo)adjuvant
LPRL	None	None	Leuprolide (ODB-GB)	Adjuvant	(neo)adjuvant
PACL(W)+TRAS[b]	Trastuzumab	Paclitaxel	None	Adjuvant	(neo)adjuvant
PERT+TRAS	Trastuzumab	None	Pertuzumab (none)	Adjuvant	(neo)adjuvant
TMXF	None	None	Tamoxifen (ODB-GB)	Adjuvant	(neo)adjuvant
TRAS[b]	Trastuzumab	None	None	Adjuvant	(neo)adjuvant
TRIP	None	None	Triptorelin (ODB-GB)	Adjuvant	(neo)adjuvant

[a](neo)adjuvant implies both the neoadjuvant and adjuvant settings;
[b]Trastuzumab (biosimilar) funded under the NDFP; Trastuzumab (Herceptin) funded under the Evidence-Building Program
[c]Funded by New Drug Funding Program during the study period, but at the time of writing it became funded by the Systemic Treatment Quality-Based Procedures
DD – dose-dense; W – weekly; ODB – Ontario Drug Benefits; GB – general benefits; EAP – exceptional access program; CA – compassionate access

© 2022 Habbous S et al. *JAMA Network Open.*

All regimens listed are for adjuvant/curative intent and can be found here: https://www.cancercareontario.ca/en/drugformulary/regimens

© 2022 Habbous S et al. *JAMA Network Open.*

eTable 2: Number of patients receiving each systemic therapy regimen by time period and type of treatment

Disease site	Regimen[b,c]	Pre-COVID era (March 11, 2019 to March 10, 2020)			COVID era (March 11, 2020 to September 30, 2020)			Neoadjuvant or non-surgical management in COVID vs. pre-COVID era[a]
		Neoadjuvant	Adjuvant	Systemic only	Neoadjuvant	Adjuvant	Systemic only	OR (95% CI)
Breast	All chemotherapies[d]	1266	2329	299	700	668	231	2.07 (1.84-2.33)
	AC-PACL(DD)	751	822	125	398	232	107	2.04 (1.70-2.45)
	AC-PACL(DD)+TRAS	63	48	11	55	21	7	1.92 (1.04-3.54)
	AC-PACL(W)	77	159	35	36	43	14	1.65 (1.03-2.65)
	AC-PACL(W)+TRAS	58	70	16	21	11	8	2.49 (1.16-5.37)
	CRBP-DOCE-TRAS	53	61	10	49	19	16	3.31 (1.78-6.16)
	CYCL-DOCE	40	551	36	27	159	30	2.60 (1.77-3.82)
	CYCL-DOCE+TRAS	13	68	7	16	17	<6	3.80 (1.67-8.65)
	FEC-D	90	169	15	39	62	13	1.35 (0.87-2.10)
	FEC-D+TRAS	77	63	9	23	11	14	2.46 (1.17-5.20)
	PACL(W)+TRAS	18	234	27	19	66	14	2.60 (1.54-4.40)
Breast	Hormonal therapies[d]	108	3489	499	190	858	283	3.17 (2.75-3.65)
	Anastrazole (ANAS)	17	1076	124	23	274	76	2.76 (2.06-3.68)
	Letrozole (LETR)	64	1253	233	92	282	101	2.89 (2.31-3.61)
	Tamoxifen (TMXF)	14	1102	123	57	292	101	4.35 (3.35-5.66)

[a] comparison of non-surgical therapy starts (neoadjuvant + systemic only) versus adjuvant treatments in the COVID era relative to the pre-COVID era. Odds ratios >1 signify patients starting treatment in the COVID era are more likely than patients starting treatment in the pre-COVID era to start treatment with systemic therapy rather than surgery
[b] due to overlap between the Activity Level Reporting (ALR) database and the New Drug Funding Database (NDFP), to avoid double-counting, all numbers were derived from ALR
[c] regimen descriptions can be found on the Ontario Health (Cancer Care Ontario) Drug Formulary (https://www.cancercareontario.ca/en/cancer-treatments/chemotherapy/drug-formulary)
[d] some regimens were omitted from the table due to small counts but still contributed to the total, including breast chemotherapy regimens [AC, AC-DOCE+/-TRAS, AC-PACL+/-TRAS, AC-PACL(W)+PERT+TRAS, capecitabine, CMF(PO), CRBP-DOCE, CRBP-DOCE+PERT+TRAS, DAC, FEC-D+PERT+TRAS, FEC100, KADC, PERT+TRAS, and TRAS], breast hormonal therapies [exemestane, goserelin, leuprolide, and triptorelin]

© 2022 Habbous S et al. *JAMA Network Open.*

eTable 3: Likelihood of starting treatment with a systemic agent by region

Disease site	Region (Local Health Integration Network)	Pre-COVID era[a]		COVID era[a]		Odds ratio (95% confidence interval)[b]
		Neoadjuvant-intent ST	Adjuvant ST	Neoadjuvant-intent ST	Adjuvant ST	
**Breast**	**All chemotherapies**	**1565**	**2329**	**931**	**668**	**2.07 (1.84-2.33)**
	Central	235	282	128	94	1.63 (1.19-2.24)
	Central East	177	256	102	56	2.63 (1.80-3.84)
	Central West	118	127	68	21	3.49 (2.01-6.04)
	Champlain	94	255	94	71	3.59 (2.44-5.30)
	Erie St. Clair	73	117	41	34	1.93 (1.13-3.32)
	Hamilton Niagara	175	267	82	74	1.69 (1.17-2.44)
	Mississauga Halton	136	230	91	75	2.05 (1.41-2.98)
	North East	63	115	22	30	1.34 (0.71-2.51)
	North Simcoe Muskoka	51	68	29	27	1.43 (0.76-2.71)
	North West	34	33	16	15	1.04 (0.44-2.43)
	South East	55	94	34	28	2.08 (1.14-3.78)
	South West	88	193	61	63	2.12 (1.38-3.27)
	Toronto Central	154	149	73	50	1.41 (0.92-2.16)
	Waterloo Wellington	95	114	87	28	3.73 (2.25-6.18)
	Unknown	17	29	<6	<6	–
**Breast**	**Hormonal therapies**	**607**	**3489**	**473**	**858**	**3.17 (2.75-3.65)**
	Central	93	463	54	81	3.32 (2.20-5.00)
	Central East	120	397	69	93	2.45 (1.69-3.56)
	Central West	20	129	11	24	2.96 (1.26-6.95)
	Champlain	23	265	56	94	6.86 (4.00-11.77)
	Erie St. Clair	17	215	31	62	6.32 (3.28-12.18)
	Hamilton Niagara	63	447	39	136	2.03 (1.31-3.17)
	Mississauga Halton	50	331	32	64	3.31 (1.97-5.56)
	North Simcoe Muskoka	24	174	17	36	3.42 (1.67-7.02)
	South East	41	170	31	43	2.99 (1.68-5.31)
	South West	39	231	23	74	1.84 (1.03-3.28)
	Toronto Central	55	291	62	71	4.62 (2.96-7.22)
	Waterloo Wellington	30	181	28	34	4.97 (2.64-9.35)
	North East/North West	23	155	15	41	2.47 (1.18-5.15)
	Unknown	9	40	<6	<6	–

[a] patients started treatment in the pre-COVID era (11/Mar/2019-10/Mar/2020) or the COVID era (11/Mar/2020-31/Sept/2020)
[b] Neoadjuvant-intent in the COVID-era versus adjuvant in the pre-COVID era
ST – systemic treatment

eTable 4: Likelihood of starting treatment with a systemic agent by public health unit

Public Health Unit (PHU)	Odds ratio (95% confidence interval)[a]	Populations (2016 Census)[b]	Num of COVID cases[c] per 10,000 persons
Algoma	1.41 (0.49-4.04)	113,084	27.41
Brant County	2.72 (1.24-5.96)	134,943	146.73
Chatham-Kent	2.13 (0.77-5.90)	102,042	359.66
Durham Region	2.58 (1.71-3.88)	645,862	331.49
Eastern Ontario	5.87 (2.67-12.9)	202,762	129.22
Grey Bruce	5.43 (2.43-12.5)	161,977	79.02
Haldimand-Norfolk	1.11 (0.36-3.43)	109,652	436.84
Haliburton, Kawartha, Pine Ridge	2.65 (1.25-5.63)	179,083	128.43
Halton Region	1.65 (1.07-2.53)	548,430	211.33
Hamilton	1.95 (1.27-3.00)	536,917	210.09
Hastings Prince Edward Counties	2.86 (1.46-5.60)	161,180	34.12
Huron Perth District	4.27 (1.78-10.3)	136,093	93.32
Kingston Frontenac and Lennox & Addington	1.24 (0.55-2.76)	193,363	65.68
Lambton	1.34 (0.56-3.21)	126,638	273.22
Leeds, Grenville, Lanark district	3.52 (1.75-7.08)	169,244	227.48
Middlesex-London	1.33 (0.83-2.14)	455,526	189.67
Niagara Region	1.89 (1.19-2.98)	447,888	238.45
North Bay Parry Sound District	2.20 (0.95-5.10)	123,820	30.69
Northwestern	0.64 (0.13-3.03)	76,455	65.40
Ottawa	3.80 (2.69-5.39)	934,243	459.20
Peel	3.21 (2.36-4.37)	1,381,744	657.79
Peterborough	1.85 (0.87-3.93)	138,236	85.36
Porcupine	0.57 (0.06-5.26)	84,201	97.39
Renfrew Country and District	3.33 (1.16-9.58)	103,593	50.20
Simcoe Muskoka District	2.69 (1.76-4.12)	540,249	164.92
Southwestern	2.09 (0.86-5.07)	199,840	134.11
Sudbury & District	2.50 (1.14-5.47)	196,448	52.43
Thunder Bay District	1.29 (0.47-3.56)	151,884	69.79
Timiskaming	1.00 (0.15-6.64)	33,049	54.46
Toronto	2.35 (1.92-2.89)	2,731,571	658.05
Waterloo	4.66 (2.98-7.28)	535,154	319.35
Wellington-Dufferin-Guelph	2.91 (1.48-5.70)	284,461	227.10
Windsor-Essex County	3.55 (2.21-5.72)	398,953	664.24
York Region	2.31 (1.71-3.12)	1,109,909	377.87
Unknown	2.56 (1.09-6.00)	113,084	27.41

[a] Neoadjuvant-intent in the COVID-era versus adjuvant in the pre-COVID era

[b] Populations (2016 Census) data by PHU was captured from Statistics Canada website on June 8th, 2021. https://www12.statcan.gc.ca/census-recensement/2016/dp-pd/prof/search-recherche/lst/results-resultats.cfm?Lang=E&TABID=1&G=1&Geo1=&Code1=&Geo2=&Code2=&GEOCODE=35&type=0

[c] Number of COVID cases by PHU was captured from Government of Ontario website, "Daily change in cases by PHU.csv" on November 3rd, 2021. https://data.ontario.ca/dataset/status-of-covid-19-cases-in-ontario

© 2022 Habbous S et al. *JAMA Network Open.*

eFigure 1: The distribution of times (months) from the Ontario Cancer Registry diagnosis date until the first systemic treatment date

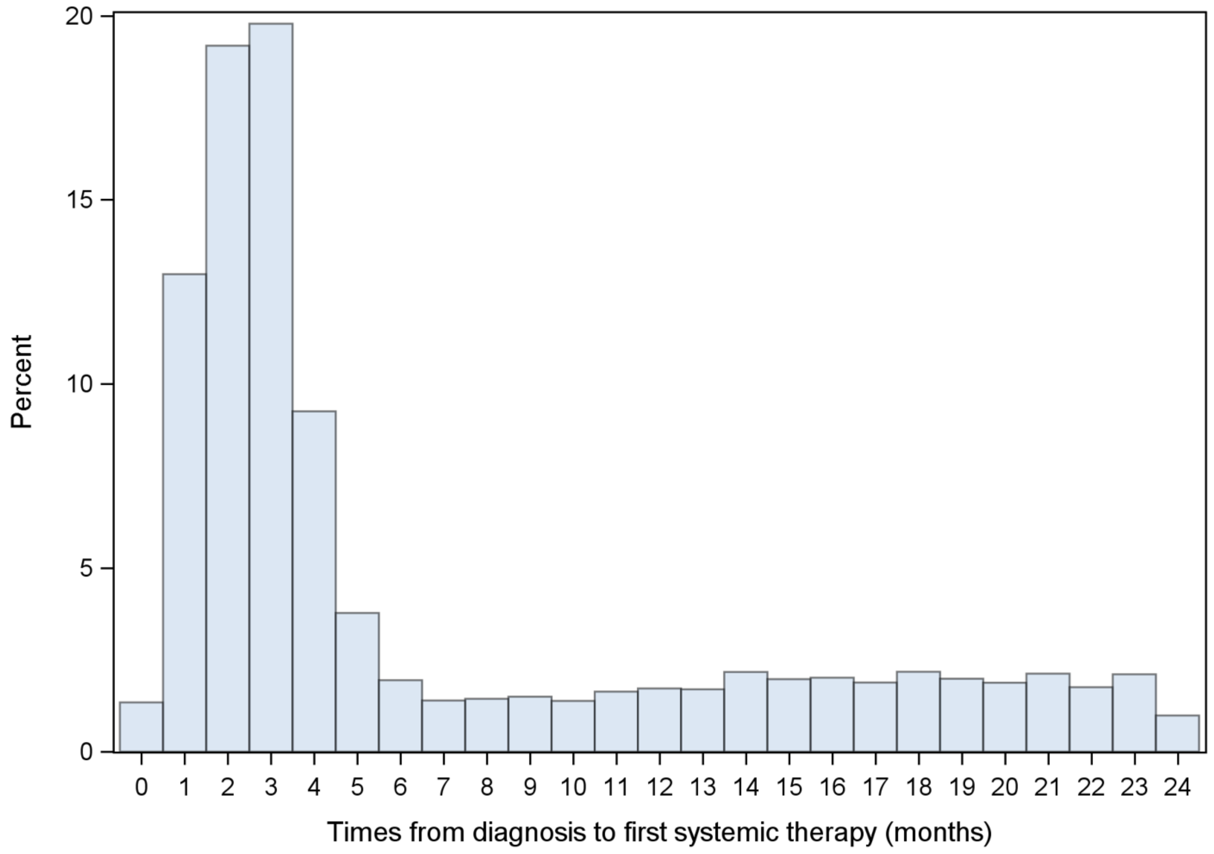

© 2022 Habbous S et al. *JAMA Network Open.*

eFigure 2: The distribution of times (days) from the start of systemic treatment until receipt of surgery for patients receiving systemic treatment first

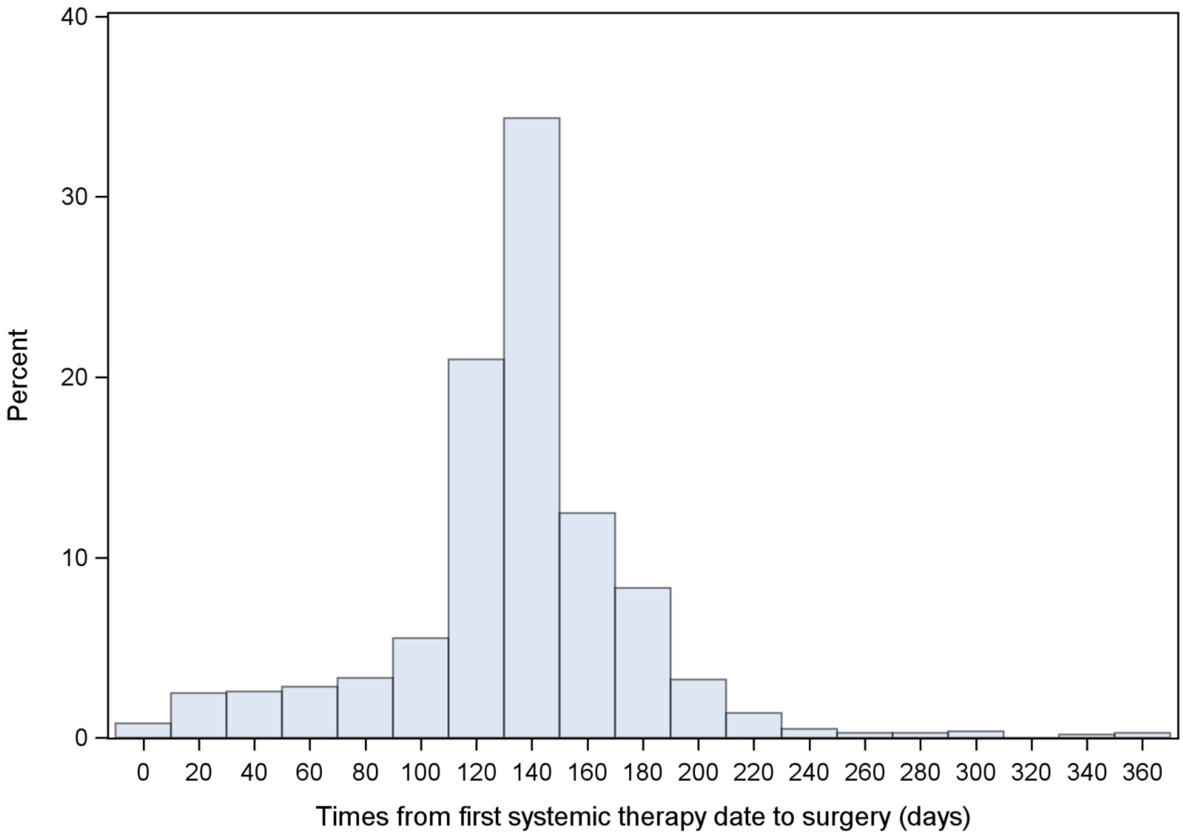

© 2022 Habbous S et al. *JAMA Network Open.*

eFigure 3: Relative odds of starting treatment with systemic treatment in the COVID era versus the pre-COVID era with non-palliative intent

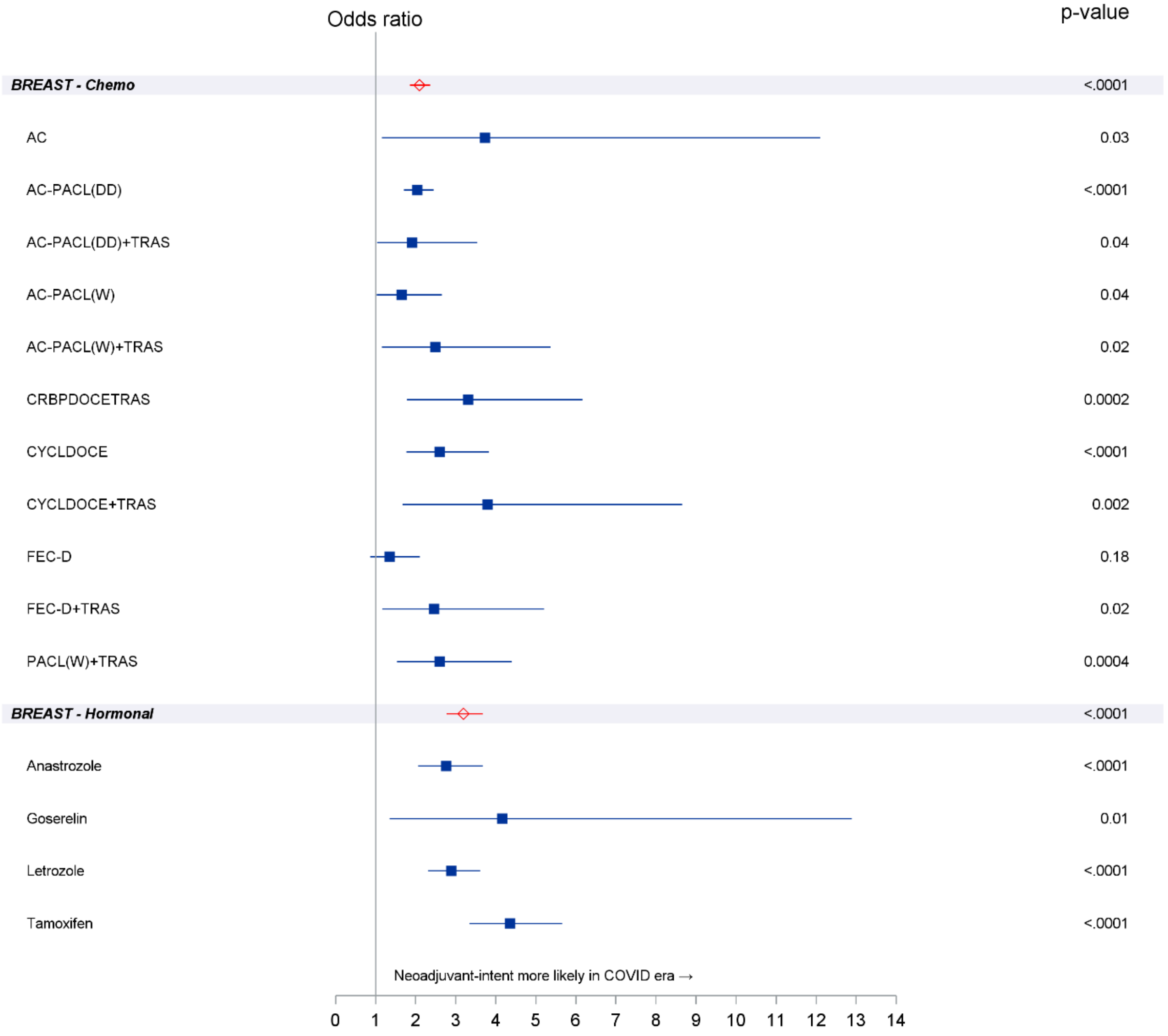

For patients with breast cancer, the most common chemotherapy regimen was dose-dense AC-paclitaxel [AC-PACL(DD)] (Table 2). The proportion of patients who received neoadjuvant-intent AC-PACL(DD) increased from 876/1698 (52%) in the pre-COVID era to 505/737 (69%) in the COVID era, corresponding to an OR 2.04 (1.70-2.45). Similar results were observed for patients receiving AC-PACL with or without trastuzumab (TRAS) in both the dose-dense and weekly protocols [OR 1.92 (1.04-3.54) for AC-PACL(DD)+TRAS; OR 1.65 (1.03-2.65) for AC-PACL(W); and OR 2.49 (1.16-5.37) for AC-PACL(W)+TRAS] (Figure ▲). Breast cancer patients were also

© 2022 Habbous S et al. *JAMA Network Open.*

more likely to receive neoadjuvant-intent chemotherapy in the COVID era than the pre-COVID era across a variety of other regimens, with ORs ranging from 1.35 to 3.80 [e.g. CRBPDOCE+TRAS, CYCLDOCE (+/-TRAS), FEC-D+TRAS, or weekly paclitaxel +TRAS]. Similar results were observed for hormonal therapies, including anastrazole [OR 2.76 (2.06-3.68)], letrozole [OR 2.89 (2.31-3.61)] and tamoxifen [OR 4.35 (3.35-5.66)].

© 2022 Habbous S et al. *JAMA Network Open.*

eFigure 4: Risk of COVID infection by patient group

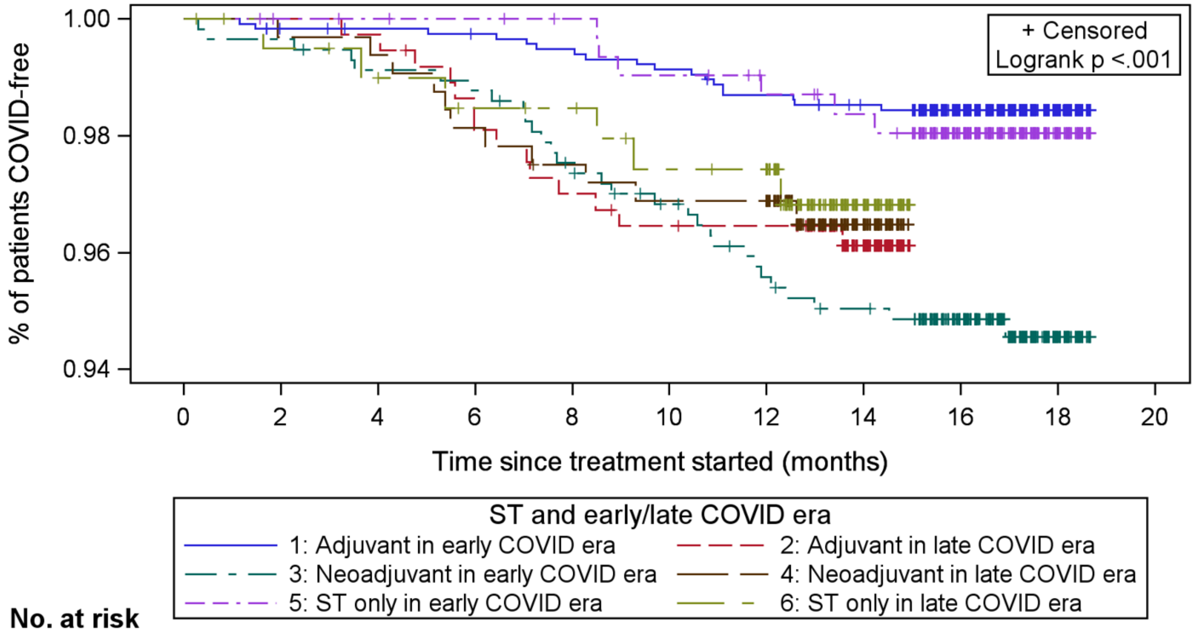

No. at risk

1	1158	1153	1151	1149	1146	1142	1136	1130	917	289	0
2	368	368	367	360	356	353	352	220	0		
3	569	567	563	561	552	544	535	530	438	92	0
4	321	320	319	315	312	310	304	100	0		
5	315	313	312	311	309	306	302	299	221	60	0
6	199	196	194	191	190	186	178	67	0		

The likelihood of COVID positivity by treatment type in the COVID era. Patients received neoadjuvant systemic treatment (ST), adjuvant ST, or ST alone. Treatment started in the early COVID era, defined as treatments starting between March 11, 2020 and June 30, 2020. Treatments starting in the late COVID era started between July 1, 2020 and September 30, 2020.

© 2022 Habbous S et al. *JAMA Network Open.*

eFigure 5: All-cause mortality by patient group

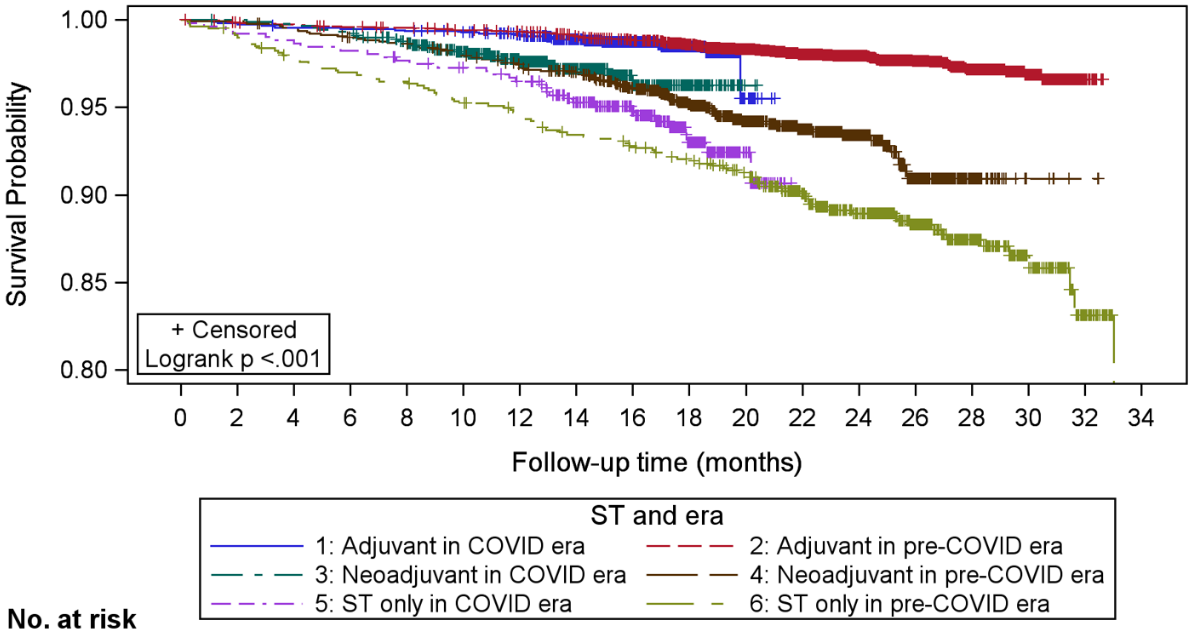

No. at risk

1	1526	1523	1518	1517	1515	1509	1487	1374	958	477	24	0					
2	5818	5805	5796	5786	5776	5763	5750	5716	5675	5541	5123	4201	3175	2177	1292	496	26	0
3	890	889	886	882	860	782	592	380	173	63	2	0					
4	1374	1373	1366	1355	1350	1337	1322	1300	1240	1053	824	602	383	191	46	6	2	0
5	514	510	508	505	500	494	488	459	357	203	66	0					
6	798	790	777	771	767	757	747	737	729	718	698	606	483	365	240	124	47	0

Patients received neoadjuvant systemic treatment (ST), adjuvant ST, or ST only. Time 0 started on the date of the first ST visit for those receiving adjuvant ST or ST alone or on the date of surgery for patients receiving neoadjuvant ST.

© 2022 Habbous S et al. *JAMA Network Open.*
